# Development of a Body Condition Scoring Index for Female African Elephants Validated by Ultrasound Measurements of Subcutaneous Fat

**DOI:** 10.1371/journal.pone.0093802

**Published:** 2014-04-09

**Authors:** Kari A. Morfeld, John Lehnhardt, Christina Alligood, Jeff Bolling, Janine L. Brown

**Affiliations:** 1 Smithsonian Conservation Biology Institute, National Zoological Park, Front Roya, Virginia, United States of America; 2 The National Elephant Center, Fellsmere, Florida, United States of America; 3 Disney's Animal Kingdom, Bay Lake, Florida, United States of America; University of Florida, United States of America

## Abstract

Obesity-related health and reproductive problems may be contributing to non-sustainability of zoo African elephant (*Loxodonta africana*) populations. However, a major constraint in screening for obesity in elephants is lack of a practical method to accurately assess body fat. Body condition scoring (BCS) is the assessment of subcutaneous fat stores based on visual evaluation and provides an immediate appraisal of the degree of obesity of an individual. The objective of this study was to develop a visual BCS index for female African elephants and validate it using ultrasound measures of subcutaneous fat. To develop the index, standardized photographs were collected from zoo (n = 50) and free-ranging (n = 57) female African elephants for identifying key body regions and skeletal features, which were then used to visually determine body fat deposition patterns. This information was used to develop a visual BCS method consisting of a list of body regions and the physical criteria for assigning an overall score on a 5-point scale, with 1 representing the lowest and 5 representing the highest levels of body fat. Results showed that as BCS increased, ultrasound measures of subcutaneous fat thickness also increased (*P*<0.01), indicating the scores closely coincide with physical measures of fat reserves. The BCS index proved to be reliable and repeatable based on high intra- and inter-assessor agreement across three assessors. In comparing photographs of wild vs. captive African elephants, the median BCS in the free-ranging individuals (BCS = 3, range 1–5) was lower (*P*<0.001) than that of the zoo population (BCS = 4, range 2–5). In sum, we have developed the first validated BCS index for African elephants. This tool can be used to examine which factors impact body condition in zoo and free-ranging elephants, providing valuable information on how it affects health and reproductive potential of individual elephants.

## Introduction

Efforts to maintain a self-sustaining population of African elephants (*Loxodonta africana*) in zoos have met with limited success [1], in part because of high mortality and low reproduction; over the past decade, there have been only three births to five deaths annually in the U.S. [2]. If this trend continues, the captive population will pass through a bottleneck in 30 years, and could become demographically nonviable in about 50 years [2]. Contributing to this problem is a dramatic increase in the rate of ovarian cycle problems, which in African elephants has increased from 25% to 46% in just 7 years [3]. To prevent further population declines, the Association of Zoos and Aquariums Elephant Taxon Advisory Group/Species Survival Plan (AZA TAG/SSP) Management Committee has endorsed research to better understand causes of poor health and reproduction of African elephants [4]. One condition that is suspected of having a significant effect on both of these problems is excessive body weight [5], [6].

Studies in horses (often used as a model for elephants) and women show that being overweight can lead to biological changes related to metabolic conditions (i.e., insulin resistance), arthritis, foot problems and impaired fertility, and that many of these can be alleviated by implementing weight reduction programs (e.g., reduced caloric intake and/or exercise) [7], [8], [9], [10]. One problem in zoo elephants is poor foot health and arthritis [5], [6], [11], [12], with a strong inverse relationship existing between foot pathology and levels of exercise [12]. Ovarian acyclicity also is a serious reproductive problem in captive African elephants, and a previous study found a relationship between a high body mass index and ovarian acyclicity in this species [13]. Circumstantial evidence suggests there may be a link between obesity-related changes and poor health and reproduction in elephants [5], [6], [12].

A major constraint in screening for obesity in African elephants is the lack of a practical method to accurately assess body condition. Although scales for direct body weights and body measurement techniques to predict body weight are available for use in elephants [14], [15], these methods do not differentiate body weight due to fat or muscle, and therefore are limited in their use for assessing degree of fatness. Standard methods for measuring body fat in humans are impractical for elephants, as they rely on potentially stressful, costly and invasive manipulations (e.g., heavy water dilution, hydrostatic weighing) [16], [17]. Skinfold calipers are widely used in humans, but with conflicting results, and these have not yielded good results in elephants [18]. An alternate method used in veterinary medicine is body condition scoring (BCS), a subjective assessment of subcutaneous body fat stores based on visual or tactile evaluation of muscle tone and key skeletal elements [19], [20]. BCS methods rely on a numeric scoring system, with higher scores representing animals with more body fat. A variety of BCS systems have been developed for farm, exotic, and research animal management [19], [21], [22] including an 11-point scale for Asian elephants [18].

Ultrasound measures of actual fat thickness have been used to validate BCS methods in a number of domestic and non-domestic species, including cattle [22], moose (*Alces alces*) [23], elk (*Cervus elaphus*) [24], mule deer (*Odocoileus hemionus*) [25] woodland caribou (*Rangifer tarandus caribou*) [26], and pinnepeds [27], [28], [29]. The AZA Standards for Elephant Management and Care [30] recommends the BCS system of Wemmer et al. [18] be used for routine assessment of body condition in Asian elephants, although it has yet to be validated ultrasonographically. However, the AZA does not currently recommended a BCS method for assessing body condition in African elephants. Given the health and reproductive problems facing African elephants in zoos [3], [5], [6], [12], [13], [31], [32] especially the high rate of ovarian acyclicity, a reliable and validated method to assess body condition for this species is needed.

The objective of this study was to develop a simple, reliable method to score body condition in African elephants that can be applied using either direct observations or the assessment of standardized photographic views, and validate it by ultrasound measures of subcutaneous fat. Ultimately, the goal is to develop a validated BCS index that may be used to determine if excessive body weight is related to morbidity, mortality or infertility in zoo African elephants.

## Materials and Methods

### Body condition scoring index

**Figure 1:**
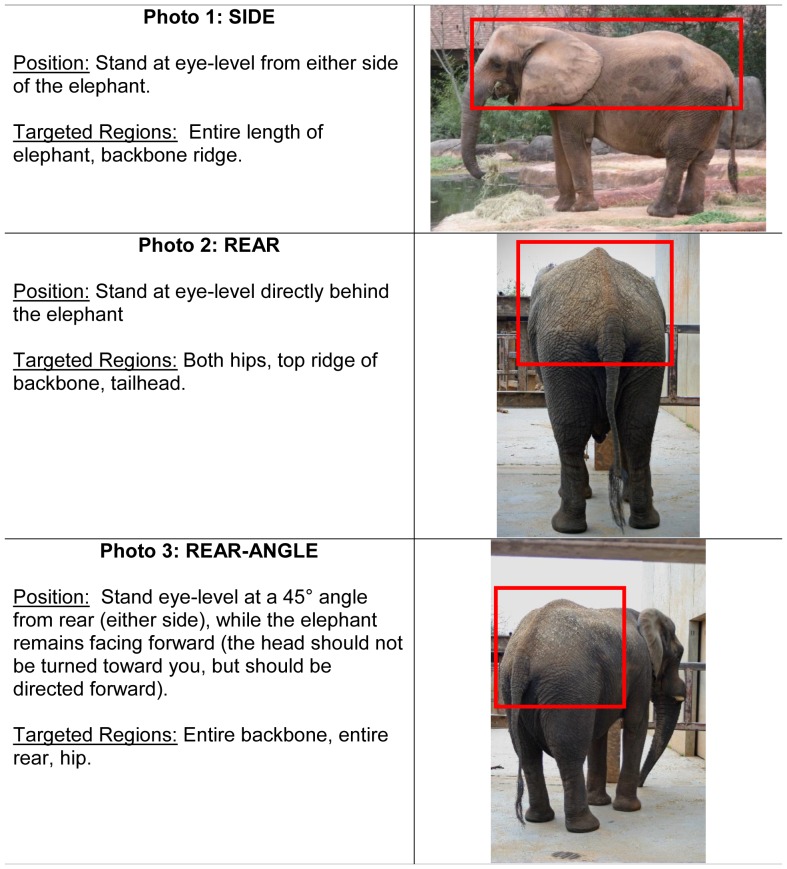
Guide for taking standardized photographs for body condition scoring.

**Figure 2:**
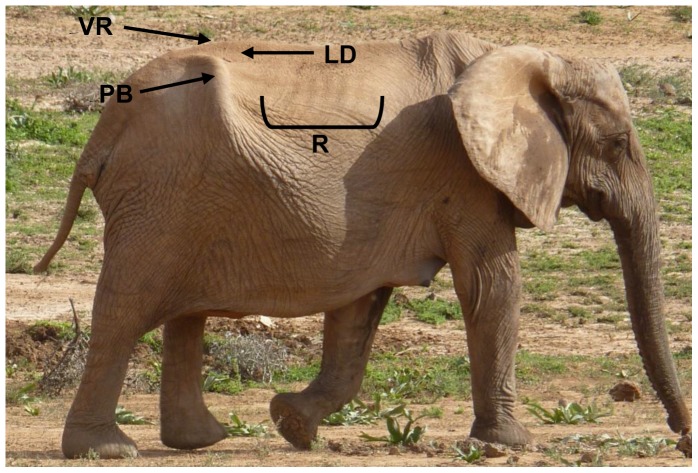
Key areas for visually assessing body condition in female African elephants. R =  ribs, PB =  pelvic bone, VR =  vertebral ridge of backbone, LD =  lumbar depression alongside backbone.

**Figure 3:**
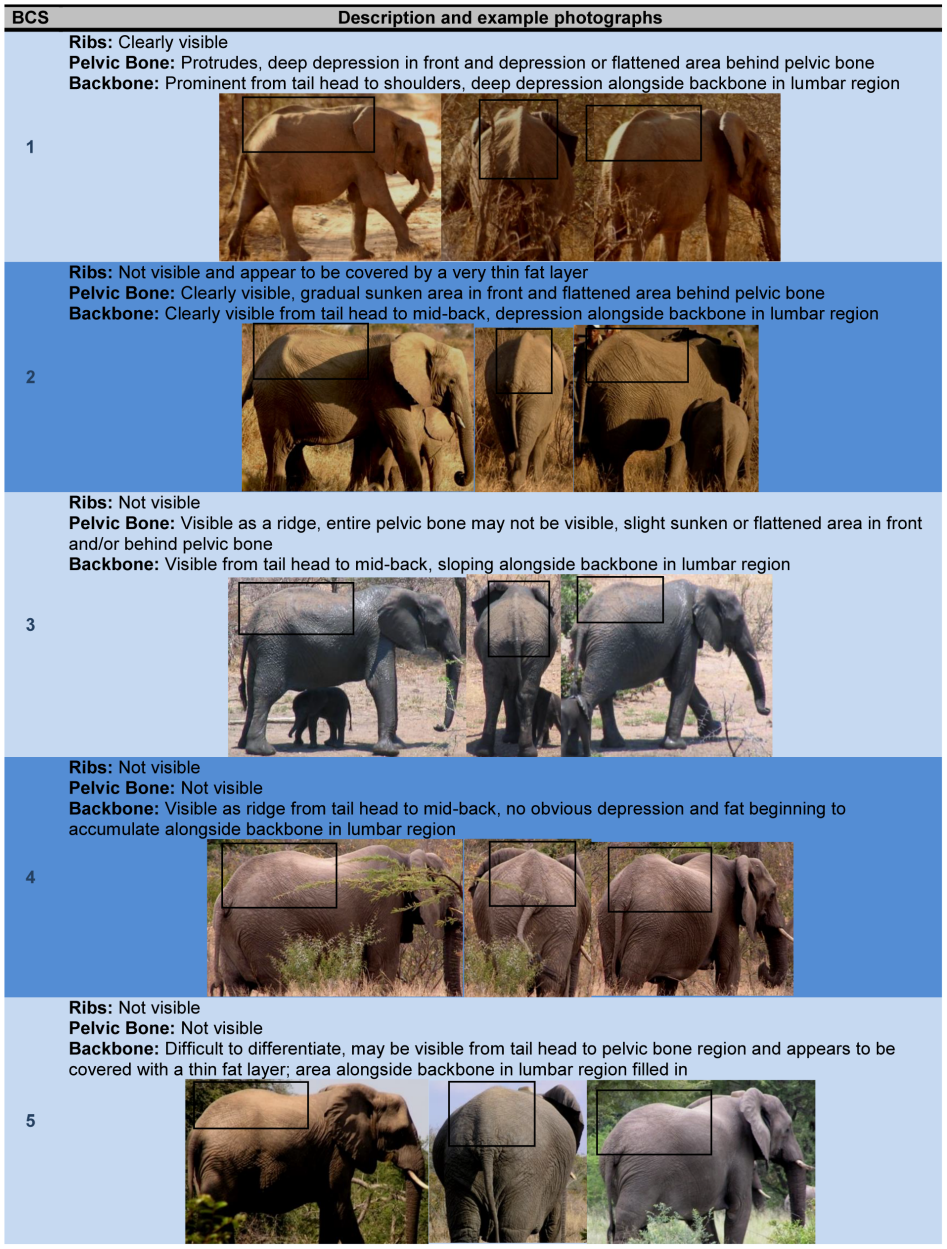
Body condition scoring index for female African elephants.

**Figure 4:**
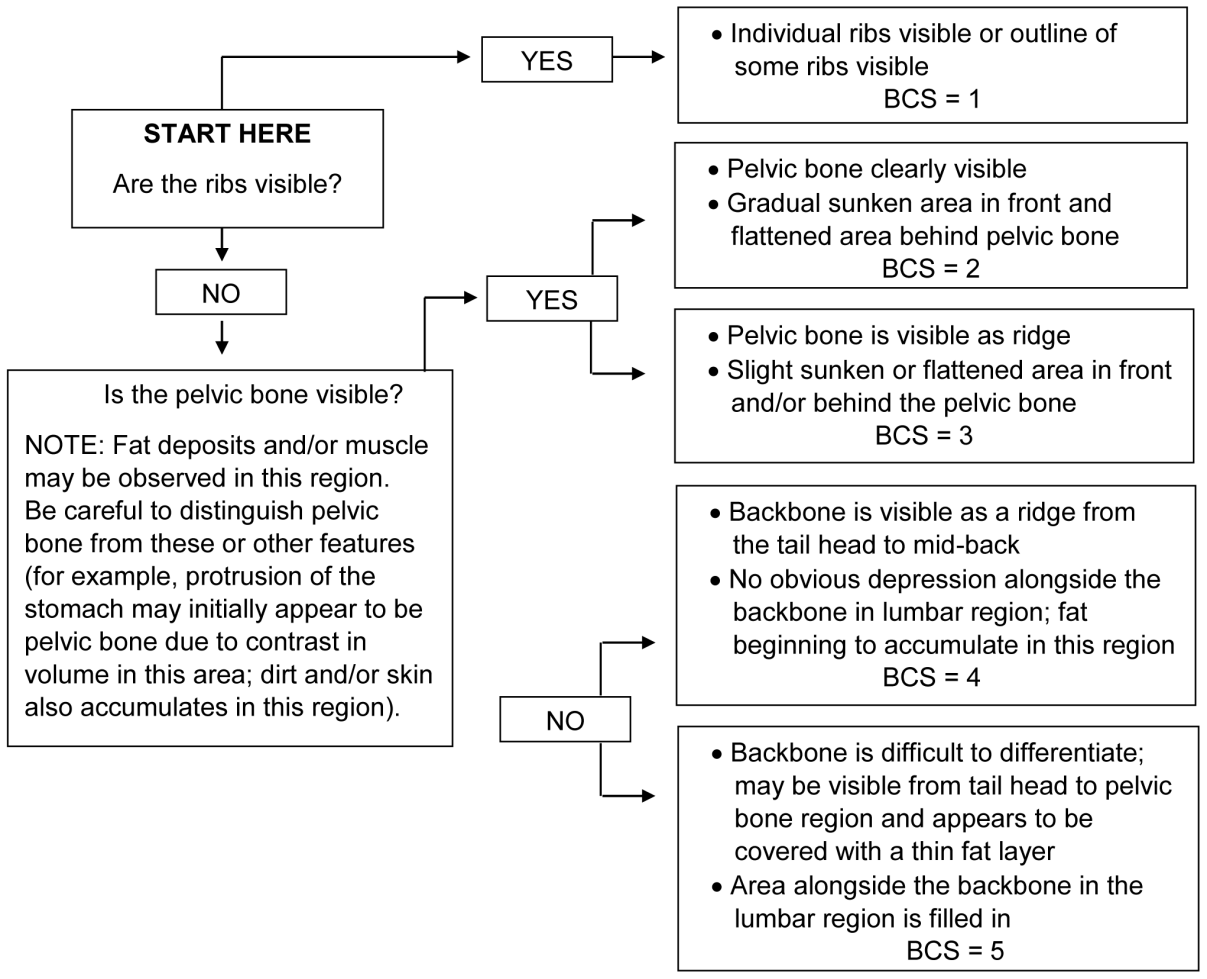
Body condition scoring flow chart for female African elephants.

This study was approved by Institution Animal Care and Use Committees of the Smithsonian Conservation Biology Institute (SCBI) and all participating zoos. The study was also approved by the Associated Private Nature Reserves and South African National Parks in 2003. Photographs of elephants have been taken as part of an on-going elephant identification study in South Africa. A total of 107 elephants were utilized in the development of the BCS index, about half (n = 50; age range, 10–45 years) of which were housed in AZA-accredited zoos. Each participating institution submitted a series of photographs taken by zoo staff using a “Photographing Guide” that provided detailed instructions for obtaining a set of three standardized photographs of each elephant from three angles (side view, rear view, and rear-angle view) (Figure 1). To compare the body condition of captive vs. free-ranging African elephant females, similar sets of photographs from a photographic dataset at South Africa's Kruger National Park (KNP), Tanda Tula Camp were examined (n = 57; age range, 10–45 years). Photographs of free-ranging elephants were age matched and chosen randomly from the database to provide a representative sample without bias for comparison to the captive population. Using photographs from captive and free-ranging elephants, key body areas were identified to serve as the anatomical regions for assessing body fat deposition patterns (Figure 2). From these, the BCS Index was created (Figure 3), as was a BCS Flow Chart (Figure 4) to systematically guide an assessor through the scoring process.

### Ultrasound measures of subcutaneous fat

A subset of 33 captive adult female African elephants at 12 AZA-accredited zoos was used for ultrasound measures of subcutaneous fat thickness to validate the visual BCS index. Anatomical regions for assessing body fat reserve depositions were: 1) directly along the lumbar vertebrae (vertebral ridge); 2) lumbar depression alongside the lumbar vertebrae (lumbar depression); 3) directly on the iliac crest of ilium (pelvic bone); 4) in front of the iliac crest of ilium (pelvic bone); and 5) behind the iliac crest of ilium (pelvic bone) (Figure 2). From a preliminary study, the ‘ribs’ were found to be difficult to locate consistently, especially in elephants with more fat reserves, and so were not included in the ultrasound validations.

An ultrasonographic unit (A-scan mode) (Preg-Alert Pro, Renco Corporation, MN, USA) was used to measure subcutaneous fat thickness. This equipment was specifically calibrated for use in elephants to measure fat depth up to 20 cm. Ultrasound measurements were obtained by one individual (KM) throughout the study. To perform the exam, ultrasound gel was applied to the desired location and five measurements were obtained at each body region. The mean thickness of the subcutaneous fat layer was calculated for each location and an overall mean of the five body regions for each elephant was calculated. Ultrasound measurements were obtained for either the right or left side for regions involving the pelvic bone and lumbar depression, whereas vertebral ridge measurements were obtained at the body midline. Ultrasound assessments (n = 5 each) were conducted in the following order: 1) vertebral ridge – the length of the lumbar region of the vertebrae column, spanning a length of approximately 25 cm; 2) lumbar depression - 2–3 cm to either side of the lumbar region of vertebrae column, spanning a length of approximately 25 cm; 3) pelvic bone - probe oriented directly on the iliac crest and moved along the entire length; 4 and 5) in front of and behind the pelvic bone, respectively - probe was placed 2 to 3 cm on either side (front or behind) of the iliac crest and moved around these regions spanning a length of approximately 13 cm. Measurements were obtained by holding the transducer for 2–3 seconds in each location until the subcutaneous fat layer measurement (in mm) was displayed.

### Reliability study

To determine inter-assessor and intra-assessor reliability of the BCS method, three assessors scored sets of photographs (side view, rear view, and rear-angle view) in a pilot study of 40 captive African elephants. Raters included a graduate student that developed the BCS index (Assessor A), an elephant specialist who has worked with elephants for >30 years and contributed to the development of the Asian elephant BCS index [18] (Assessor B), and an animal behaviorist with no prior experience in scoring body condition of any species (Assessor C). Each assessor independently scored the photographic sets twice within a 1-month period. Assessors remained blinded to the previous scores and were not permitted to discuss study results.

### Statistical methods

To compare BCS between free-ranging and captive elephants, the Cochran-Mantel-Haenszel statistic for linear trend was used to test the null hypothesis that BCS was randomly distributed between the free-ranging and captive groups. The five ultrasound measurements were averaged to provide a mean ultrasound measurement of fat thickness for each body region, and an overall mean for all body regions was also calculated. The relationships between BCS and ultrasound measurements were investigated using regression analyses. Spearman correlation coefficients were used to determine correlations among BCS and ultrasound measurements of the five body regions. Correlations were considered to be different from zero at *P*<0.01.

For the reliability study, the overall percentage (%) agreement between paired intra- and inter-assessor assessments was calculated as (100×*m*)/*n*, where *n* =  total number of samples examined and *m* =  number of cases of exact agreement. A weighted kappa (κ*_w_*) statistic was also used to analyze intra- and inter-assessor variability [33]. Standards proposed by Lanidis and Koch [34] were used to interpret resulting kappa values, where perfect agreement equates to a kappa of 1 and chance agreement equates to 0. The following standards for interpreting kappa values for strength of agreement were used: kappa values ≤0 =  poor, 0.10 to 0.20 = slight, 0.21 to 0.40 =  fair, 0.41 to .60 =  moderate, 0.61 to 0.80 =  substantial and 0.81 to 1.00 =  almost perfect agreement.

## Results

### Ultrasound validation

**Figure 5:**
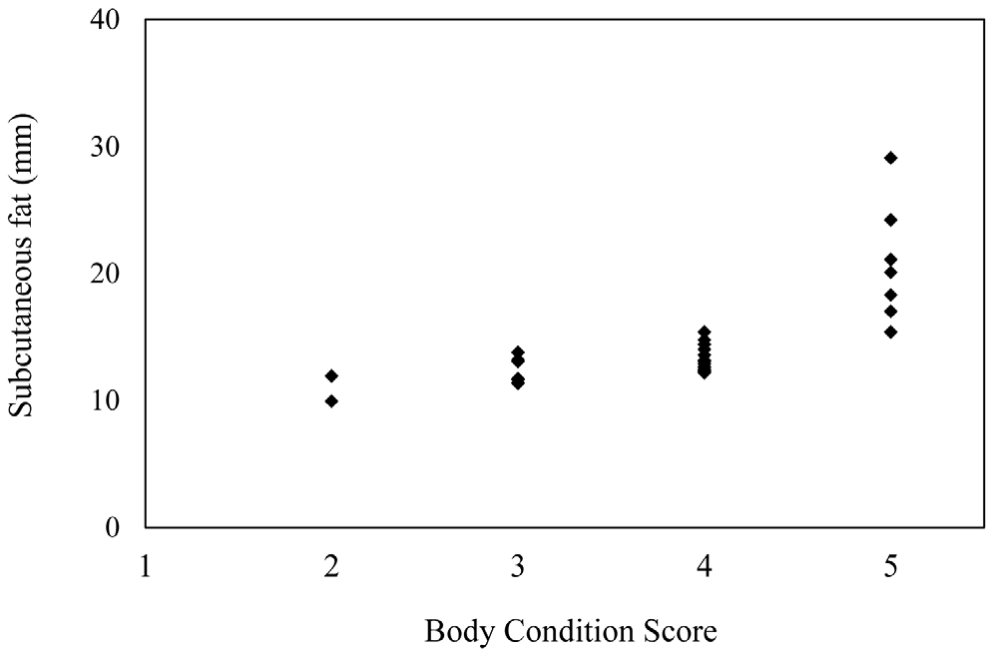
Scatter plot of body condition scores and overall mean ultrasound measurements of fat thickness for all five body regions combined in female African elephants.

Table 1 shows the ultrasound measurements of subcutaneous fat thickness for each body region and the mean of all five regions for elephants of different BCSs. Ultrasound measurements of fat thickness at all five body regions and the means of the five regions were significantly associated with BCS (Table 1, *P*<0.001). As the BCS increased, the fat thickness also increased (*P*<0.001), indicating that BCS adequately reflected the amount of fat reserves. Spearman correlation coefficients for correlation of BSC with specific body regions and mean of the five regions evaluated by ultrasound are shown in Table 2. There were positive correlations (*P*<0.01) between the BCS and mean ultrasound measures of fat thickness at each body region, and for the overall mean of the five regions. Of the five specific body regions, the vertebral ridge had the strongest correlation to BCS (r = 0.748, *P*<0.0001), and the mean of the five regions showed an even stronger correlation (r = 0.816, *P*<0.0001). A scatter plot of BCS and mean ultrasound measurement of fat thickness for the five body regions is presented in Figure 5, which shows that the mean ultrasound measures of fat thickness increased as BCS increased.

**Table 1:** Mean (SD) ultrasound measurements of subcutaneous fat thickness by body region and body condition score (BCS) category in zoo African elephants (N = 33).

	Subcutaneous fat thickness (mm)					
BCS^1^	Vertebral ridge	Lumbar depression	Pelvic bone	Pelvic bone-front	Pelvic bone-back	Mean^2^
2 (N = 2)	9.90 (1.41)	10.90 (0.14)	11.50 (2.40)	11.20 (1.31)	11.10 (1.56)	10.92 (1.41)
3 (N = 8)	12.25 (1.41)	12.33 (2.04)	12.08 (1.35)	12.15 (1.21)	12.10 (1.74)	12.18 (0.99)
4 (N = 15)	12.83 (1.55)	13.06 (1.56)	13.55 (2.07)	13.80 (2.18)	13.69 (2.75)	13.39 (1.13)
5 (N = 8)	21.75 (7.46)	20.80 (6.99)	20.03 (5.28)	22.20 (6.63)	19.08 (6.34)	20.77 (4.33)
*P*-value	0.0003*	0.0003*	0.007*	0.0023*	0.0007*	0.0008*

1 BCS (1 = lowest to 5 = most body fat); no zoo elephant had a BCS = 1.

2 Mean subcutaneous fat thickness of all body regions.

*Resulting *P*-value from regression analyses with each body region and the means of all body regions analyzed separately.

**Table 2:** Spearman correlations among body condition score (BCS) and mean ultrasound measurements of fat thickness of specific body regions.^1^

	BCS	VR	LD	PB	PB-F	PB-B	Mean
**BCS**	1.000	0.748*	0.707*	0.665*	0.745*	0.658*	0.816*
**VR**		1.000	0.615*	0.549*	0.657*	0.623*	0.791*
**LD**			1.000	0.555*	0.717*	0.490*	0.794*
**PB**				1.000	0.725*	0.562*	0.769*
**PB-F**					1.000	0.615*	0.897*
**PB-B**						1.000	0.809*
**Mean**							1.000

1 VR = vertebral ridge, LD =  depression alongside lumbar region of backbone, PB = directly on pelvic bone, PB-F = in front of pelvic bone, PB-B = behind pelvic bone.

Mean = mean of all body regions.

*Significant (*P*<0.01).

### Reliability study

Intra-assessor reliability: The level of percentage agreement ranged from 88–95% between repeat BCS assessments of the photograph set of 40 captive elephants for the three assessors (Table 3). Weighted kappa values ranged from 0.8–0.9, and the interpretation of κ*_w_* values for repeat assessment for all assessors was “excellent”, according to the criteria of Landis and Koch [34].

**Table 3:** Level of intra-and inter-assessor agreement for assessment of elephant body condition.

	Intra-assessor agreement			Inter-assessor agreement		
**Assessor**	A	B	C	A and B	A and C	B and C
**Percentage (%) agreement**	95	90	88	93	73	73
**κ** ***_w_*** ** (95% CI)**	0.93 (0.88–0.98)	0.81 (0.74–0.88)	0.82 (0.78–0.86)	0.89 (0.82–0.96)	0.67 (0.58–0.76)	0.62 (0.52–0.72)

κ*_w_* =  weighted kappa; 95% CI = 95% confidence interval.

Inter-assessor reliability: The level of percentage agreement for assigning a BCS to the set of 40 elephants among assessors ranged from 73%–93%, with the greatest agreement between assessors A and B (Table 3). Weighted kappa values for assessments between assessors A and B were interpreted as “almost perfect” agreement, whereas all other inter-assessor agreements were interpreted as “substantial” agreement when applying the methods of Landis and Koch [34].

### Body condition scoring of captive and free-ranging elephants

Body condition scores differed between captive and free-ranging elephants, with a trend towards higher body condition scores for captive elephants and a trend towards lower body condition scores for free-ranging elephants (Table 4, *P* = 0.0001). The median BCS for elephants in the captive population (n = 50) was a 4 (range 2–5, Table 4), which was higher than that for free-ranging elephants (n = 57) (BCS = 3, range 1–5, Table 4). The most prevalent (mode) BCS observed in the free-ranging study population was a 2 (39%, n = 22), lower than the mode BCS = 5 (40%, n = 20) observed for the captive population. None of the captive elephants had a BCS = 1, whereas 9% (n = 5) of the free-ranging elephants scored in that category.

**Table 4:** Body condition scores (BCS) (and relative percentage) of free-ranging and captive female African elephants in each BCS category.

	Free-ranging elephants	Captive elephants	
BCS	Number (%)	Number (%)	*P*- value
1	5 (9)	0 (0)	
2	22 (39)	2 (4)	
3	18 (33)	12 (24)	
4	10 (18)	16 (32)	
5	2 (4)	20 (40)	
**Total**	57	50	
**Median (range)**	3 (1–5)	4 (2–5)	
**Mean (SD)**	3.74 (0.96)	4.39 (0.58)	0.0001*

*Cochran-Mantel-Haenszel test for linear trend.

## Discussion

We have developed a new visual BCS index for assessing body fat and condition in female African elephants. The scoring method consists of a list of body regions and the physical criteria used for assigning an overall score on a 5-point scale, with 1 being the least and 5 being the most body fat. The index includes example photographs of elephants representing each BCS, and a ‘Body Condition Scoring Flow Chart’ to systematically guide an assessor through the scoring process when using direct observations or sets of standardized photos. This is the first BCS system for African elephants that has been validated by ultrasound measures of actual body fat, and was found to be a simple, reliable assessment tool.

A BCS index (1–6 scale) was previously described for African elephant bulls [35]; however, that publication did not include photographs representing each body condition category and only brief anatomical descriptions were provided. By contrast, the Wemmer index for Asian elephants [18] included numerous photographs of elephants at each BCS, but was based on a rather extensive scoring system - six regions of the body scored using two or three criteria per region, and then the six scores totaled to obtain an overall score ranging from 0–11 (where 0 is the thinnest). Our goal was to simplify the Wemmer technique by reducing the number of scoring criteria, and to validate the method with direct measures of fat thickness using ultrasound, something that was not done by Poole [35] or Wemmer [18]. First, we excluded several body regions (e.g., head and shoulder regions) because no consistent variations in fat thickness were observed in these areas, especially for elephants with a higher BCS. Second, the Wemmer BCS index requires observing the entire elephant from all angles and adding the scores from specific body regions, which can be difficult under field conditions. By contrast, our new BCS index does not require observing the head or shoulder regions, and an overall BCS can be assigned cumulatively by assessing only the backbone, pelvic bone, and rib areas.

The new BCS index was validated with ultrasound measures of subcutaneous fat thickness, and significant positive correlations were observed at all five body regions. As the BCS increased, subcutaneous fat thickness increased, similar to that described for other species [25], [27], [28], [36], [37], [38], [39]. These findings show that appropriate body regions were identified for developing the new BCS index in African elephants. The backbone region (vertebral ridge) provided the highest correlation to the BCS (r = 0.748, *P*<0.001, Table 2), although all correlations were statistically significant. Thus, any of the designated body regions could be used as a single validated indicator of body condition. In humans, certain anatomical regions (e.g., the abdominal region) give a better prediction of overall body fat than others [40], [41] and the same has been found for cattle [22], horses [42] and dogs [39]. Recently, ultrasound was used to validate a new visual 9-pt BCS index for Asian elephants [43]. That study assessed ultrasonic fat thickness in the rump region only (n = 12), whereas we evaluated ultrasound fat thickness at multiple body regions. Nevertheless, results were comparable in both studies in showing a significant relationship between measured fat thickness and BCS. The median ultrasonic measure of subcutaneous rump fat in the Asian elephants was 1.07 cm (range, 0.41–3.11 cm) and the median BCS was 6.25. These findings are in accordance with our measurements of fat thickness at the most comparable region assessed (directly behind the pelvic bone), which had a median fat thickness of 1.31 cm (range, 1.0–3.28 cm). Our ultrasound fat thickness measures observed in elephants with high BCS (∼2.5 cm) were also comparable to the highest fat thickness reported in other herbivore species, including cattle (∼3.5 cm) [22], horses (∼3.1 cm) [38], elk (∼3.7 cm) [24], [26], deer (∼3.5 cm) [24], and moose (∼7 cm) [23], [24].

The new BCS index proved to be a reliable method for assessing female African elephant body condition based on high intra- and inter-assessor agreement across three assessors. Determining the accuracy of a new diagnostic test can be difficult when there is no accepted reference or “gold” standard, and none of the previous elephant BCS studies tested assessor reliability. Two types of reliability exist: agreement between assessments made by two or more assessors (inter-assessor reliability); and agreement between assessments made by the same assessor on two or more occasions (intra-assessor reliability). Agreement can be measured using several statistics: percent agreement to provide an overall agreement rate; and the kappa statistic, which is a measure of agreement that indicates the proportion of agreement expected by chance. A weighted kappa was used to reflect the degree of disagreement so that a greater emphasis was placed on large differences between or among assessments compared to small differences, which is commonly adopted in ordinal scale reliability assessments [33]. The resulting weighted kappa value was then interpreted in terms of the strength of agreement among assessments. Landis and Koch [34] proposed the following as standards to determine the strength of agreement in reliability studies: kappa values ≤0 = poor, 0.1–0.20 =  slight, 0.21–0.40 =  fair, 0.41–0.60 =  moderate, 0.61–0.80 =  substantial and 0.81–1 =  almost perfect agreement. Similar formulations exist to interpret weighted kappa results for reliability studies, but with slightly different descriptors. For example, an alternate scale was developed by Fleiss [44], where kappa value <0.41 =  poor, <0.75–0.41 =  good - fair, and >0.74–1.0 =  excellent agreement. We chose the formulation of Landis and Koch [34] because it is often cited in studies assessing reliability in body condition scoring systems [45], [46]. Regardless of the scale used, kappa values above 0.40 are considered to be clinically useful [47], and all assessments in our study had values above this clinically relevant level. The current study demonstrated high percentage agreement (73% to 95%) and an overall “substantial” strength of agreement determined by the weighted k statistic (κ*_w_* = 0.62 to 0.91) between and among assessors using the new BCS index.

Body condition scoring has become an integral part of assessing body fat in veterinary practices because it is an effective and inexpensive way to quantify patient condition. In other species, BCS indexes are used to categorize an individual in terms of body fat (i.e. thin, normal, overweight, obese), and within the same species various BCS methods may be employed with the middle score commonly representing the “ideal” or “normal” body condition. For example, two numeric scales are typically used and accepted in veterinary practices for assessing body condition in dogs (5-pt and 9-pt scales) [48], [49]. When using a 5-point scale, the “ideal/normal” BCS = 3, BCS = 1–2 equates to “underweight/thin” and “overweight/obese” includes BCS = 4–5. When using a 9-point scale, the “ideal/normal” BCS = 4–5, whereas “underweight/thin” is represented by BCS = 1–3 and “overweight/obese” include BCS = 6–9. Finally, in cattle, both 9-point [50], [51] and 5-point [52], [53] scales are used, and the middle scores represent the “ideal” distribution of body fat. Based on these criteria, a BCS = 4 or 5 in elephants using our 5-point scale would equate to “overweight/obese” categories, whereas a BCS = 3 would indicate an “ideal/normal” body condition. If this classification holds, 40% (n = 20) of the elephants in our zoo study were obese, differing from the free-ranging elephants where only 4% (n = 2) of the study population had a BCS = 5. Furthermore, 32% (n = 16) of zoo elephants could be classified as overweight (BCS = 4), whereas only 18% (n = 10) of free-ranging elephants scored in this category. Last, only 12 zoo elephants (24%) were in “ideal” condition (BCS = 3) and two (4%) were “thin” (BCS = 2), compared to 72% of wild African elephants that had a BCS of 2 or 3. We used the free-ranging population in South Africa's Kruger National Park (KNP), Tanda Tula Camp as a reference or benchmark because the elephants are protected, and in general the habitat quality is good [54], [55]. In addition, Save the Elephants at Tanda Tula has an existing and extensive photographic database on hundreds of elephants. Photographs were chosen randomly and across seasons with standard views to match those of the captive population study. While additional scoring of wild African elephants certainly is needed to define optimal or suboptimal body condition status under varied conditions, the data are beginning to suggest that captive elephants are on the higher end of the scale, and thus may not represent the “ideal/normal” condition. Further investigations also are warranted to define elephant obesity in terms of associated health complications, similar to what has been done in horses, humans, and other species [9], [56], [57], [58], [59], [60]. This new BCS index should enable better monitoring of body condition in individual and populations of elephants, allowing for more directed medical and management decisions based on scientific data and validated tools. For example, managers can monitor the effects of changes in diet formulations or exercise programs on BCS in conjunction with actual weight measurements to optimize zoo husbandry practices and promote a more healthy and sustainable population.

Ultimately, our goal was to develop an easy and reliable means of assessing BCS because of the need to investigate possible relationships between obesity and health and reproductive problems in zoo-managed elephants [6], [11], [61]. It is important to recognize that the new BCS method assesses subcutaneous fat rather than visceral fat. Visceral obesity, or a disproportionate amount of body fat within the abdominal cavity, is linked to the pathogenesis of obesity related diseases such as cardiovascular disease and type 2 diabetes in humans [62], [63]. The distribution of body fat, for example intra-abdominal fat, rather than total body fat may be important to consider when investigating relationships with obesity-related health concerns in elephants. The new BCS method includes example photographs of elephants representing each BCS category and a flow chart to systematically guide an assessor through the body condition scoring process. The relationships between BCS and ultrasound measurements determined that scores adequately reflect the amount of subcutaneous fat of elephants, and that most zoo elephants are in the higher condition categories. The observation that 30% of zoo-managed African elephants maintain consistent baseline levels of progestagens, indicative of complete ovarian inactivity, and another 16% exhibit irregular cycles is a serious concern, especially because over three quarters of abnormal cycling females are of reproductive age [3]. The relationship between a high body mass index (BMI) and ovarian acyclicity in African elephants [13], suggests that reproductive problems may be caused in part by metabolic derangements associated with excessive body fat in this species. This seems possible given studies in horses and humans indicate obesity can lead to metabolic changes that are correlated with impaired fertility [60], [64], [65], [66], [67], [68]. Our new elephant BCS index is a quick and reliable tool that has been validated to assess body condition in female African elephants for routine health monitoring. By instituting obesity screening of zoo elephants, facilities can monitor and potentially identify at-risk elephants in time to mitigate health and reproductive problems.
